# Cadmium und seine anorganischen Verbindungen – Addendum: Reevaluierung des BLWs: Beurteilungswerte in biologischem Material

**DOI:** 10.34865/bb744043d10_4ad

**Published:** 2025-12-22

**Authors:** Ernst Hallier, Hans Drexler, Andrea Hartwig, MAK Commission

**Affiliations:** 1 37136 Ebergötzen Deutschland; 2 Friedrich-Alexander-Universität Erlangen-Nürnberg. Institut und Poliklinik für Arbeits-, Sozial- und Umweltmedizin Henkestraße 9–11 91054 Erlangen Deutschland; 3 Institut für Angewandte Biowissenschaften. Abteilung Lebensmittelchemie und Toxikologie. Karlsruher Institut für Technologie (KIT) Adenauerring 20a, Geb. 50.41 76131 Karlsruhe Deutschland; 4 Ständige Senatskommission zur Prüfung gesundheitsschädlicher Arbeitsstoffe. Deutsche Forschungsgemeinschaft, Kennedyallee 40, 53175 Bonn, Deutschland. Weitere Informationen: Ständige Senatskommission zur Prüfung gesundheitsschädlicher Arbeitsstoffe | DFG

**Keywords:** Cadmium, Biologischer Leitwert, BLW, Nephrotoxizität, Urin, urine

## Abstract

The German Senate Commission for the Investigation of Health Hazards of Chemical Compounds in the Work Area (MAK Commission) re-evaluated the data for cadmium [7440-43-9] to derive a biological guidance value (BLW) for its systemic non-carcinogenic end points. Relevant studies were identified from a literature search. Exposure to cadmium dust can cause nasal inflammation and anosmia, bronchitis and pneumonia, which are recognised as local effects. Tubular kidney damage was identified as the most sensitive end point of systemic toxicity, which results in excretion of low molecular weight proteins such as α1 and β2-microglobulins and retinol binding protein (RBP) in the urine. Recent studies on workers occupationally exposed to cadmium revealed a NOEL (no observed effect level) and a BMDL5 (benchmark dose lower limit) for tubular proteinuria at approximately 3 to 5 µg cadmium/g creatinine in ever-smokers, whereas this threshold is higher in never-smokers. Therefore, a BLW of 2 µg cadmium/g creatinine is set for cadmium in urine.

**Table:** 

**BLW (2024)**	**2 µg Cadmium/g Kreatinin**
**BAR (2010)**	**1 µg Cadmium/l Blut** ^a)^ **0,8 µg Cadmium/l Urin** ^a)^
	Probenahmezeitpunkt: keine Beschränkung im Fließgleichgewicht
	
**MAK-Wert**	**–**
Hautresorption (2004)	H
Krebserzeugende Wirkung (2004)	Kategorie 1

^a)^ für Raucher gelten andere Werte

## Reevaluierung

Cadmium wurde im Jahr 2004 in die Kategorie 1 der krebserzeugenden Arbeitsstoffe eingestuft (Greim 2004). Hierfür maßgeblich war die Verursachung von Lungentumoren durch Inhalation von Cadmiumverbindungen und auch Tumorbefunde an den Nieren wurden berichtet. Eine Konzentration, bei der keine kanzerogene Wirkung beobachtet wurde, konnte nicht bestimmt werden. Ein Biologischer Arbeitsstoff-Toleranzwert (BAT-Wert) kann daher nicht abgeleitet werden. An den Atemwegen und der Lunge verursachen cadmiumhaltige Stäube und Dämpfe akute und subchronische Entzündungen („Cadmiumschnupfen“ und Anosmie, Bronchitis, Pneumonie), die in erster Linie lokale Effekte sind.

Hinsichtlich systemischer Wirkungen von Cadmium stehen die Nieren im Vordergrund. Die Nieren akkumulieren Cadmium bei chronischer Exposition und sind daher sowohl Haupt-Speicherorgan als auch kritisch gefährdetes Zielorgan. Insbesondere werden die Nierentubuli und bei höheren Belastungen auch die Glomeruli geschädigt. Aufgrund der gestörten Rückresorption in den Nierentubuli werden vermehrt Calcium und Phosphat mit dem Urin ausgeschieden; dadurch kann es zur Osteomalazie mit Spontanfrakturen der Knochen (in den 1950er Jahren als „Itai-Itai-Krankheit“ beschrieben) und zu einer Bildung von Harnsteinen kommen. Eine zusätzliche Osteoporose mit Verlust von Knochenmatrix (reduzierte Knochendichte) wird berichtet. Frauen sind hinsichtlich der Knochenschäden durch Cadmium besonders gefährdet, u. a. aufgrund der postmenopausalen Osteoporose, eines erhöhten Calciumverlustes durch das Stillen, einer geringeren Proteinproduktion des Metallothioneins sowie durch Eisenmangel, der eine erhöhte intestinale Aufnahme von Cadmium bewirkt.

Eine arbeitsbedingte Exposition gegen Cadmium und seinen anorganischen Verbindungen erfolgte in früheren Jahrzehnten bei der Beschichtung von Metallen zum Korrosionsschutz (Verkadmierung) und der Verarbeitung solcher beschichteten Metalle (Schweißen, Trennen, Schneiden). Der quantitativ größte Einsatz erfolgte in der Herstellung von Nickel-Cadmium-Akkumulatoren bzw. -Batterien, insbesondere wiederaufladbaren Batterien, die in den 1990er und 2000er Jahren mehr als 80 % des weltweiten Einsatzes von Cadmium ausmachten. Des Weiteren kommen Cadmiumverbindungen für Pigmente in der Fotoindustrie und Fotovoltaik, in der Herstellung von Spezialglas (z. B. Fernsehbildschirme, Ampelanlagen) und als Stabilisatoren für Kunststoffe zum Einsatz. Da Cadmium in der Batterieherstellung mittlerweile durch andere Metalle ersetzt wurde, ist die industrielle Bedeutung dieses Metalls und seiner Verbindungen erheblich zurückgegangen; eine arbeitsbedingte Gefährdung besteht heute in erster Linie bei der Entsorgung und dem Recycling alter Batterien.

Im Jahr 2007 wurde für Cadmium ein Biologischer Leitwert (BLW) von 7 µg Cadmium/l Urin abgeleitet. Dieser basierte auf der nephrotoxischen Wirkung von Cadmium, die bei beruflicher Exposition beobachtet wurde. Damals wurde geschlossen, dass oberhalb einer Konzentration von 5 µg Cadmium/g Kreatinin (entsprechend 7,5 µg Cadmium/l Urin) eine tubuläre Nierenschädigung auftritt (Käfferlein 2008). Zudem wurde von einer Reversibilität der Proteinurie bei Belastungen unter 5 µg Cadmium/g Kreatinin bzw. unter 7,5 µg/l Urin ausgegangen.

Im Jahr 2010 wurde der BLW von 7 µg Cadmium/l Urin wieder ausgesetzt (Drexler et al. 2011). Diese Aussetzung beruhte auf Hinweisen, dass die tubuläre Proteinurie irreversibel ist und bereits eine geringere Belastung von 4 µg Cadmium/g Kreatinin zu einer glomerulären Schädigung mit verringerter glomerulärer Filtrationsrate (GFR) führen kann (Järup et al. 1995). Zudem gaben Studien über umweltbedingte Expositionen Hinweise darauf, dass eine verringerte GFR bei ähnlich niedrigen Cadmiumbelastungen auftreten kann, wie sie bei tubulären Schädigungen beobachtet werden (Åkesson et al. 2005; Suwazono et al. 2006), und dass bereits bei einer Ausscheidung von 1–2 µg Cadmium/g Kreatinin die Wahrscheinlichkeit auffällig erhöhter Werte für tubuläre Mikroproteine bei 10 % liegt (Buchet et al. 1990, 1991). Auch ist die lange Halbwertszeit mit der Akkumulation von Cadmium in den Nieren zu berücksichtigen.

Seit der letzten Evaluierung des BLWs im Jahr 2010 sind weitere Studien über beruflich und umweltbedingt Exponierte erschienen, vor allem aber wurden neue Erkenntnisse über die Pathophysiologie der Nieren veröffentlicht, die eine Neubewertung erforderlich machen.

## Pathophysiologische Aspekte der Nierentoxizität von Cadmium

### Metallothionein

Die klassische, durch zahlreiche Forschungsarbeiten in vivo und in vitro belegte Hypothese zur Cadmiumtoxizität an den Nieren besagt, dass Cadmium an das Protein Metallothionein gebunden vorliegt. Dieses niedermolekulare Protein wird überwiegend in der Leber, in geringem Maβe auch in Tubuluszellen der Nieren produziert (Klaassen et al. 2009). Nach der glomerulären Filtration gelangt es in den Primärharn, wird im proximalen Tubulus rückresorbiert und reichert sich dort an. Über die Zeit degeneriert das Metallothionein, so dass Cadmium wieder freigesetzt wird und lokal toxisch wirkt. Die Folge ist eine Tubulusschädigung der Niere, und infolge der gestörten physiologischen Rückresorption werden vermehrt Elektrolyte (u. a. Calcium, Phosphat) und niedermolekulare Proteine wie α1-Mikroglobulin (α1MG), β2-Mikroglobulin (β2MG), Retinolbindendes Protein (RBP) und *N*‑Acetyl-β-D-glucosaminidase (NAG) mit dem Endharn ausgeschieden. Diese Mikroproteine werden als Marker der Tubulopathie laboranalytisch erfasst. Dabei wird die obere Normgrenze für β2MG im Spontanurin in der deutschen klinischen Literatur mit 0,3 mg/l bzw. 0,2 bis 0,3 mg/g Kreatinin angegeben (Gressner und Arndt 2019). Die kontinuierliche Freisetzung von Cadmium aus dem Metallothionein und dessen Konzentration im Endharn korreliert mit der Gesamtlast des in der Nierenrinde gespeicherten Cadmiums. Die Cadmiumkonzentration im Urin wird deshalb als Maβ für die chronische Cadmiumbelastung angesehen.

Bei gesunden Erwachsenen im typischen Arbeitsalter trifft diese Hypothese zu. Problematisch ist die Situation hingegen bei einer glomerulären oder interstitiellen Nierenschädigung, z. B. bei einer diabetischen Nephropathie oder einer chronischen Glomerulonephritis. Metallothionein hat eine hohe Affinität zum Cadmium, jedoch bindet das Metall auch weniger spezifisch an diverse andere Proteine (Fels et al. 2019), insbesondere, wenn aufgrund einer Glomerulopathie die Gesamteiweiß-Ausscheidung (Albumin) erhöht ist. Zudem ist die Cadmiumhomöostase von anderen, um die Eiweißbindung konkurrierenden Metallen abhängig, insbesondere vom Eisen- (Thévenod und Wolff 2016) und Zinkhaushalt (Nordberg und Nordberg 2022).

### Alter und Kreatininbezug

Ein zu berücksichtigender Aspekt bei der Ableitung eines BLWs ist das Alter, da die geringere Kreatininausscheidung von Personen älter als 60 oder 70 Jahre, evtl. mit chronischen Erkrankungen, unvermeidlich zu einer statistischen Assoziation zwischen höheren kreatininadjustierten Cadmiumkonzentrationen und einer (pathologischen) Proteinurie führt. Jedoch ist diese Assoziation nicht darauf zurückzuführen, dass Cadmium ein hohes Alter oder chronische Erkrankungen verursacht, sondern darauf, dass eine hohe adjustierte Cadmiumkonzentration aus einer verminderten Kreatininausscheidung resultiert. Auch Satarug et al. (2021) weisen auf die Problematik des Kreatininbezugs hin und fordern eine Bestimmung der Kreatininclearance. Anhand ihrer Studiendaten wiesen sie nach, dass der Effekt des Cadmiums auf die GFR durch Normierung der Ausscheidungsrate über die Kreatininclearance aufgehoben wird.

Ebenfalls problematisch ist die Einbeziehung von Heranwachsenden, insbesondere in den populationsbasierten umweltepidemiologischen Studien. Bei Volumenbezug sinkt die Cadmiumkonzentration im Urin in der Jugend auf ein Minimum von ca. 0,2 µg/l bei 20-jährigen und steigt dann bis zu einem Maximum im Alter von 60 bis 70 Jahren an (Chaumont et al. 2013). Bei Kreatininbezug hingegen haben Kinder und Heranwachsende Cadmiumkonzentrationen im Urin, die ähnlich oder sogar höher sind als bei Erwachsenen, obwohl die Körperlast des Metalls 5- bis 10mal niedriger ist (Bernard 2016).

### „Reverse causation“

Bei Vorliegen nicht beruflich bedingter Gesundheitsstörungen mit Auswirkungen auf die Nierenfunktion und die Proteinausscheidung kann die Dosis-Wirkungs-Beziehung zwischen der Cadmiumkonzentration und der Ausscheidung tubulärer Markerproteine verfälscht werden. Dieses Phänomen ist von verschiedenen Autoren als „reverse causation“ beschrieben worden, die vor allem bei niedrigen Cadmiumbelastungen als Störeffekt (Confounder) wirkt (Åkesson et al. 2014; Bernard 2016; Buser et al. 2016; Vacchi-Suzzi et al. 2016 a, b).

Die positive Assoziation zwischen der Ausscheidung niedermolekularer Proteine und der Cadmiumkonzentration im Urin ist bei Nie-Rauchern schwächer als bei Rauchern (Åkesson et al. 2014). Bernard (2016) wies darauf hin, dass in Umweltstudien Cadmium als Kausalfaktor für Wachstumsstörungen, kindliche Entwicklungsstörungen, Knochenentmineralisierung und Frakturen, Nierenfunktionsstörungen und -erkrankungen, Fertilitätsstörung, Diabetes, Bluthochdruck etc. bereits bei Cadmiumkonzentrationen von < 0,5 µg/g Kreatinin angesehen wird. Dies impliziert, dass Cadmium bei niedriger Belastung toxischer ist als bei hoher (Bernard 2016). Bei Nierenspendern mit einer Cadmiumkonzentration im Urin im Median von 12,9 µg Cadmium/g Kreatinin ist aber keine Beeinträchtigung der Glomerulärfunktion zu verzeichnen (Wallin et al. 2016).

Buser et al. (2016) berichteten über die Ergebnisse der bevölkerungsbezogenen NHANES-Erhebung in den USA zum Zusammenhang zwischen Cadmium und Blei in Blut und Urin und der Nierenfunktion. NHANES habe eine inverse Assoziation zwischen GFR und Cadmium im Blut beschrieben bei positiver Assoziation zwischen GFR sowie Albumin im Urin mit der Cadmiumkonzentration im Urin bei niedriger Cadmiumbelastung. „Reverse causality“ besage, dass eine reduzierte GFR die Gesamtfiltration chemischer Substanzen reduziert und in der Folge zu verminderten Stoffspiegeln im Urin und erhöhten Spiegeln im Blut führt. Eine verminderte glomeruläre Filtration bewirkt also eine Steigerung von Cadmium im Blut bei gleichzeitiger Verminderung der Urinausscheidung von Cadmium.

Byber et al. (2015) erstellten ein systematisches Review zum Zusammenhang zwischen Cadmium oder Cadmiumverbindungen und Nierenerkrankungen bei beruflich exponierten Arbeitern und in der Allgemeinbevölkerung. Hierzu wurden Studien zu 34 exponierten Kollektiven, die insgesamt mehr als 3000 Personen umfassten, ausgewertet. Die systematische Auswertung ergab keine Evidenz für eine Progression der primär tubulären Nierenschädigung durch Cadmium zu einer chronischen Niereninsuffizienz (reduzierte GFR).

### Reversibilität

Wie bereits bei Drexler et al. (2011) ausgeführt, untersuchten Järup et al. (1995) die Beziehung zwischen der Exposition gegen Cadmium und der GFR bei 42 Lötern mit mindestens fünfjähriger beruflicher Cadmiumbelastung. 24 Arbeiter zeigten eine tubuläre Proteinurie mit einer β2MG-Ausscheidung von > 34 µg/mmol Kreatinin (> 304 µg/g Kreatinin) und 17 Arbeiter mit einer β2MG-Ausscheidung von > 60 µg/mmol Kreatinin (> 536 µg/g Kreatinin). Bei 12 Arbeitern, deren GFR während der Studie dreimal gemessen worden war (1984, 1989 und 1993), wurde selbst Jahre nach dem Ende der beruflichen Exposition keine Verbesserung der GFR beobachtet.

Roels et al. (1997) untersuchten die Entwicklung der Cadmium-induzierten renalen tubulären Dysfunktion bei Arbeitern in Abhängigkeit vom Schweregrad der Mikroproteinurie bei 32 in der Vergangenheit hoch gegen Cadmium exponierten Arbeitern in zwei Beobachtungszeiträumen (1980–1984 und 1990–1992). Bei β2MG-Konzentrationen im Urin nicht über 300 μg/g Kreatinin war das Risiko gering, später eine tubuläre Dysfunktion zu entwickeln, auch in Fällen mit historischen Cadmiumkonzentrationen im Urin von gelegentlich > 10, aber immer < 20 μg/g Kreatinin. Es ergab sich ein Hinweis auf eine reversible tubulotoxische Wirkung von Cadmium, wenn eine leichte Mikroproteinurie vorlag (β2MG im Urin > 300 aber < 1500 μg/g Kreatinin) und die Cadmiumkonzentrationen im Urin nie über 20 μg/g Kreatinin lagen. Bei Vorliegen einer schweren Mikroproteinurie (β2MG im Urin > 1500 μg/g Kreatinin) und historischen Cadmiumkonzentrationen im Urin von mehr als 20 μg Cadmium/g Kreatinin war die Cadmium-induzierte tubuläre Dysfunktion trotz Reduzierung oder Beendigung der Cadmiumexposition progressiv.

Trzcinka-Ochocka et al. (2002) untersuchten 58 seit mindestens einem Jahr nicht mehr gegen Cadmium exponierte Beschäftigte einer Fabrik für Nickel-Cadmium-Batterien, die 1983 und 1986–1988 im geometrischen Mittel gegen 23,3 bzw. 55,7 µg Cadmium/l Blut exponiert waren, um die Reversibilität der Dysfunktion der Nierentubuli in Abhängigkeit vom Schweregrad der Mikroproteinurie, der Cadmiumkonzentration im Urin und der Zeit seit dem Ende der Exposition zu bewerten. Die Beschäftigten wurden entsprechend ihrer Konzentrationen von RBP im Urin in den Jahren 1986–1988 in drei Gruppen eingeteilt: < 300 (n = 26), 301–1501 (n = 25) und > 1501 µg/g Kreatinin (n = 7). Im Jahr 1999 lagen die RBP-Werte bei 85 %, 64 % bzw. 42 % dieser Personen unter 300 µg/g Kreatinin. Es ergaben sich Hinweise, dass auch im Falle einer relativ hohen früheren Exposition gegen Cadmium die tubuläre Proteinurie und möglicherweise auch der Rückgang der glomerulären Filtrationsrate reversibel sein kann.

In der Längsschnittstudie von Gao et al. (2016) an 41 nichtrauchenden Arbeiterinnen einer Fabrik zur Herstellung von Nickel-Cadmium-Batterien betrug die Nachbeobachtungszeit nach Expositionsende im Median 8 Jahre (1–10 Jahre). Über ein Regressionsmodell wurde eine Abnahme der Cadmiumkonzentration im Urin von im Mittel 3,0 µg/g Kreatinin in 10 Jahren abgeschätzt. Die Konzentrationen von β2MG und RBP schwankten relativ zu ihren jeweiligen Ausgangskonzentrationen.

## Beziehung zwischen innerer Belastung und Beanspruchung

### Berufliche Belastung

Ding et al. (2011) untersuchten 103 Eisenbahn-Schweißer (19 ♂, 84 ♀) in China mit einer beruflichen Expositionsdauer von 2 bis 21 Jahren (12,8 ± 7,27). Die Cadmiumkonzentrationen in der Luft am Arbeitsplatz betrugen 8–86 µg/m^3^ und überstiegen bei 17 % der Messungen den dortigen Grenzwert von 10 µg/m^3^. Angaben zum Tabakkonsum fehlen in der Studie. Es wurden Cadmiumkonzentrationen im Urin von 0,05 bis 12,4 µg/g Kreatinin gemessen. Schweißer mit mehr als 3 µg Cadmium/g Kreatinin hatten statistisch signifikant höhere β2MG-Werte als solche mit weniger als 3 µg/g Kreatinin. In der Tabelle 1 der Publikation sind wahrscheinlich Mittelwerte und Standardabweichungen angegeben, genaue Informationen hierzu fehlen. Auffällig sind die nicht plausiblen Standardabweichungen. Die beiden höchsten β2MG-Konzentrationen betrugen 920 und 935 µg/g Kreatinin. Die Autoren schlussfolgerten, dass eine Exposition gegen Schweißrauche zu erhöhten Werten für Cadmium im Urin und Markern der Tubulusschädigung führen kann. Weitere klinische Untersuchungen wurden nicht durchgeführt.

Die belgische Arbeitsgruppe von Haddam et al. (2011) untersuchte in Algerien 184 gesunde männliche Arbeiter einer Zinkhütte (n = 132) und 52 Beschäftigte einer Fabrik zur Herstellung von Acrylfaser-Decken. Die medianen Cadmiumkonzentrationen lagen bei den Arbeitern der Zinkhütte im Blut bei 0,80 µg/l (Interquartilsabstand (IQR) 0,45–1,16) und im Urin bei 0,70 µg/g Kreatinin (IQR 0,40–1,3), bei den Beschäftigten der Deckenfabrik im Blut bei 0,66 µg/l (IQR 0,47–0,87) und im Urin bei 0,55 µg/g Kreatinin (IQR 0,40–0,90). Die 55 rauchenden Arbeiter der Zinkhütte hatten im Vergleich zu Nichtrauchern statistisch signifikant höhere Cadmiumkonzentrationen im Blut (Median 1,20 µg/l; IQR 0,80–1,70) und im Urin (Median 1,00 µg/g Kreatinin; IQR 0,70–1,37) und eine höhere Ausscheidung von RBP und Protein HC (α1-Mikroglobulin) im Urin. Das RBP im Urin korrelierte aber nur bei Nie-Rauchern statistisch signifikant mit den Cadmiumkonzentrationen im Urin (r = 0,27; p = 0,04) und im Blut (r = 0,29; p = 0,02). Diese Zusammenhänge wurden bei Ex-Rauchern (Urin: r = 0,21; p = 0,33 und Blut: r = 0,06; p = 0,15) und Rauchern (Urin: r = – 0,04; p = 0,74 und Blut: r = 0,02; p = 0,87) nicht beobachtet. Die Autoren schlussfolgerten, dass die mit dem Rauchen verbundene höhere RBP-Ausscheidung im Urin nicht durch eine höhere Cadmiumexposition erklärt werden kann und dass Rauchen einen stärkeren Einfluss auf die Cadmiumkonzentration im Urin als der Beruf hat. Insgesamt waren die Cadmiumkonzentrationen in Blut und Urin im Bereich der Hintergrundbelastung.

Die belgische Arbeitsgruppe von Chaumont et al. (2011) untersuchte eine Kohorte von 599 Beschäftigten (451 ♂, 148 ♀) mit einem mittleren Alter von 45,4 Jahren, die in vier Fabriken für Nickel-Cadmium-Batterien in Frankreich, Schweden und den USA über einen Zeitraum von durchschnittlich 18,8 Jahren arbeiteten. Es wurde untersucht, ob das Verhältnis zwischen der Proteinausscheidung im Urin und der Cadmiumkonzentration im Urin durch die Faktoren Geschlecht, Alter, Diurese und Rauchen beeinflusst wurde. Die Beschäftigten wurden anhand ihrer Cadmiumkonzentrationen im Urin in 7 Expositionsgruppen eingeteilt (≤ 1, > 1–2, > 2–3, > 3–4, > 4–6, > 6–10, > 10 µg Cadmium/g Kreatinin). Als Referenzgruppe dienten Arbeiter mit weniger als 1 µg Cadmium/g Kreatinin. Bei Berücksichtigung aller Beschäftigten waren die Odds für „anomal“ (> 95. Perzentil der Referenzgruppe) hohe Konzentrationen von RBP und β2MG im Urin in den Gruppen mit 6 bis 10 µg Cadmium/g Kreatinin und mehr als 10 µg Cadmium/g Kreatinin statistisch signifikant erhöht. Die „benchmark dose“ (BMD5) und das „benchmark dose lower limit“ (BMDL5) für eine 5%ige Erhöhung der Hintergrundprävalenz von „anomalen“ Konzentrationen von RBP und β2MP im Urin wurden mit 5,1/3,0 µg Cadmium/g Kreatinin bzw. 9,6/5,9 µg Cadmium/g Kreatinin abgeschätzt. Wenn Jemals-Raucher ausgeschlossen wurden, waren die Odds für „anomal“ hohe Konzentrationen beider Proteine im Urin nur bei Arbeitenden mit einer Cadmiumkonzentration im Urin von mehr als 10 µg/g Kreatinin erhöht (Odds Ratio 21,8; 95-%-KI 6,4–74,4 bzw. 15,1; 3,6–63,1). Für Nie-Raucher betrugen die BMD5/BMDL5 von Cadmium im Urin 12,6/6,6 bzw. 12,2/5,5 µg/g Kreatinin, während sie bei den Jemals-Rauchern 6,2/4,9 bzw. 4,3/3,5 µg/g Kreatinin betrugen. Die Autoren leiteten ein „benchmark dose lower limit“ (BMDL5) von Cadmium im Urin für eine niedermolekulare Proteinurie infolge beruflicher Exposition gegen Cadmium von 5,5 bis 6,6 µg/g Kreatinin ab. Die Autoren folgerten, dass Tabakrauch selbst bei Personen ohne Bluthochdruck oder Diabetes die Nierenfunktion schädigt. Eine verlässliche Ermittlung der Benchmark-Dosis könne nur erfolgen, wenn Jemals-Raucher ausgeschlossen würden. Eine Störung (Confounding) der Assoziation zwischen niedrigmolekularen Proteinen und der Cadmiumkonzentration im Urin sei offenbar in erster Linie auf die schädliche Wirkung des chronischen Rauchens auf die Nieren zurückzuführen.

Gao et al. (2016) führten in Shenzhen (China) eine Längsschnittstudie an 41 nichtrauchenden Arbeiterinnen einer Fabrik zur Herstellung von Nickel-Cadmium-Batterien zum Zeitpunkt der Beendigung der Cadmiumexposition und über einen Zeitraum von bis zu 10 Jahren nach Expositionsende durch. Bei Expositionsende betrug das mittlere Alter 30,8 ± 6,7 Jahre, die Beschäftigungsdauer lag bei Expositionsende bei 7,5 ± 4,2 Jahren; Urinkonzentrationen wurden gemessen für Cadmium (Median 6,19 µg/g Kreatinin; IQR 5,17–7,45); β2MG (Median 105,38 µg/g Kreatinin; IQR 76,16–190,23) und RBP (Median 71,84 µg/g Kreatinin; IQR 44,98–115,57). Die Cadmiumkonzentrationen korrelierten statistisch signifikant mit den Konzentrationen von β2MG und RBP; die Autoren bestätigten den bekannten Zusammenhang zwischen der chronischen Cadmiumbelastung und der Tubulusfunktion der Nieren. Weder das Alter noch die Beschäftigungsdauer zeigten einen signifikanten Einfluss auf die Konzentrationen von Cadmium, β2MG oder RBP.

Choi et al. (2020) untersuchten in Südkorea in einem Kleinbetrieb 10 Silber-Lötarbeiter, die im Mittel 8,5 Jahre (3–20 Jahre) beschäftigt/exponiert waren. Die Cadmiumkonzentrationen in der Luft reichten von 6 bis 15 µg/m^3^. Bei allen 10 Arbeitern lagen die Cadmiumkonzentrationen im Blut im Durchschnitt bei 21,29 µg/l (maximal 34,6 µg/l). Bei 9 Arbeitern wurden Cadmiumkonzentrationen im Urin von durchschnittlich 22,15 µg/g Kreatinin gemessen (Maximum 62,9 µg/g Kreatinin). Die β2MG-Konzentration war bei 3 Arbeitern erhöht. Die Cadmiumkonzentrationen im Urin korrelierten positiv mit dem Proteingehalt des Urins. Die Autoren schlussfolgerten, dass Cadmium bereits bei niedrigen Luftkonzentrationen toxisch wirken kann. Bei der graphischen Darstellung einer linearen Korrelation der Werte entsprachen 30 µg Cadmium/g Kreatinin etwa 0,2 mg β2MG/l Urin.

### Umweltmedizinische Studien

Im Gegensatz zu der fast ausschließlich inhalativen Aufnahme von Cadmium aufgrund beruflicher Tätigkeiten steht bei der außerberuflichen Exposition neben der Inhalation von Tabakrauch vor allem die orale Aufnahme durch die Nahrung im Vordergrund. Im Folgenden werden seit 2010 publizierte umweltmedizinische Studien zu Cadmium mit dem Endpunkt Nierentoxizität beschrieben:

Ferraro et al. (2010) werteten Daten aus der nationalen umwelt- und ernährungsmedizinischen Erhebung NHANES der USA aus. Von 5426 Probanden (48,7 % ♂, 51,3 % ♀, mittleres Alter 47 Jahre), darunter 48,8 % Raucher, wurden die Cadmiumkonzentrationen in Blut und Urin mit der Prävalenz der chronischen Niereninsuffizienz (GFR < 60 ml/min/1,73 m^2^) und der Albuminausscheidung im Urin korreliert. Die Ergebnisse wurden für Alter, Geschlecht, BMI und Ethnie adjustiert. Personen mit Cadmiumkonzentrationen > 1 µg/g Kreatinin im Urin und Personen mit > 1 µg/l im Blut waren häufiger sowohl von einer Albuminurie (OR 1,41) als auch von chronischer Niereninsuffizienz betroffen.

Suwazono et al. (2011) führten eine bevölkerungsbasierte Studie an Personen im Alter ab 50 Jahren durch (547 ♂, 723 ♀), die in einer nicht Cadmium-kontaminierten Region in Japan lebten. Die BMDL5 für β2MG bei den Männern betrug 2,6 und bei den Frauen 1,4 µg Cadmium/g Kreatinin und für die NAG bei Männern 4,1 und bei Frauen 3,1 µg Cadmium/g Kreatinin. Angaben zum Tabakkonsum fehlen.

Akerstrom et al. (2013) untersuchten in Schweden 24-h-Urinproben von 30 gesunden Nichtrauchern beider Geschlechter, Medianalter 39 Jahre. Die Cadmiumkonzentration im Urin war positiv assoziiert mit der Ausscheidung von Albumin und α1MG. Albuminurien traten bei Cadmiumkonzentrationen oberhalb 2 µg/l Urin bzw. 2,5 µg/g Kreatinin, vermehrte α1MG-Ausscheidung oberhalb einer Cadmiumkonzentration von 3 µg/l bzw. 2,9 µg/g Kreatinin auf. Intraindividuell waren die Assoziationen stärker zwischen Ausscheidungsraten und bezüglich des spezifischen Gewichts adjustierten Konzentrationen als bezüglich Kreatinin-adjustierten Konzentrationen. Die Autoren schlussfolgerten, dass bei Nichtrauchern Assoziationen zwischen Cadmium im Urin und Markern der Nierenfunktion nicht auf eine Toxizität von Cadmium, sondern auf normale Schwankungen der Diurese zurückzuführen sind.

Thomas et al. (2013) untersuchten Patienten (599 ♂, 253 ♀) in England und Schweden mit chronischer Niereninsuffizienz (GFR < 60 ml/min). Es wurde keine Assoziation zwischen der Niereninsuffizienz und der ernährungsbedingten Cadmiumbelastung (Median 0,219 µg/g Kreatinin (0,22 nmol Cadmium/mmol Kreatinin)) festgestellt.

Hu et al. (2014) untersuchten den Zusammenhang zwischen Cadmium und den tubulären Proteinmarkern β2MG und NAG im Urin bei 490 nichtrauchenden Frauen im Alter von 35 bis 54 Jahren aus zwei Landkreisen in China. Mit steigenden Cadmiumkonzentrationen nahmen die tubulären Proteine NAG und β2MG im Urin signifikant zu. Für das 90. Perzentil als Abschneidekriterium lagen die BMD5/BMDL5 für diese Effekte bei 2,08/1,41 bzw. 3,80/2,18 µg Cadmium/g Kreatinin im ersten und bei 3,34/1,91 bzw. 0,99/0,74 µg Cadmium/g Kreatinin im zweiten Landkreis. Die Autoren schlussfolgerten, dass die BMD5 für Cadmium im Urin in China ähnlich wie der Referenzwert der European Food Safety Authority von 1 µg Cadmium/g Kreatinin sind.

Ke et al. (2015 a, b) führten eine Querschnittsstudie an 6103 Probanden im Alter 35–89 Jahre (2715 ♂, mittleres Alter 60,3 Jahre; 3388 ♀, mittleres Alter 59,5 Jahre) aus 5 chinesischen Provinzen durch, in denen der Reis aufgrund der lokalen Industrie höhere Cadmiumkontaminationen aufwies. Ziel war, die Schwelle von Cadmium im Urin für Nierenfunktionsstörungen zu ermitteln. Als Effektmarker dienten die Mikroproteine β2MG (Ke et al. 2015 a) und NAG (Ke et al. 2015 b). Die ermittelten BMDL10 für β2MG betrugen 2,00 µg Cadmium/g Kreatinin bei Männern und 1,69 µg Cadmium/g Kreatinin bei Frauen (Ke et al. 2015 a). Die BMDL5 für NAG betrug 2,08 µg Cadmium/g Kreatinin bei Männern und 1,93 µg Cadmium/g Kreatinin bei Frauen (Ke et al. 2015 b). Die Autoren zogen das Fazit, dass die BMDL in diesen chinesischen Populationen deutlich niedriger seien als der von der WHO angegebene Schwellenwert von 5 µg/g Kreatinin für cadmiumbezogene renale Effekte. Die etwas niedrigeren BMDL bei Frauen würden eine größere Empfindlichkeit gegenüber Cadmium andeuten als bei Männern. Die Publikationen enthalten keine Angaben zum Tabakkonsum oder zu Begleiterkrankungen wie Diabetes.

Daneben finden sich mehrere umweltmedizinische Studien, die für die Evaluation eines BLWs nicht belastbar sind (u. a. Robles-Osorio et al. 2017; Satarug et al. 2018).

Umweltmedizinische Studien sind für die Ableitung eines BLWs nur bedingt zu verwenden, da Faktoren wie Alter, Kreatininspiegel und Erkrankungen, u. a. Nierenerkrankungen und Diabetes mellitus, schwer zu kontrollieren sind und bei der Ableitung arbeitsmedizinischer Grenzwerte eine geringere Bedeutung haben.

## Ableitung eines BLWs für Cadmium im Urin

Im Jahr 2008 wurde festgestellt, dass bei beruflicher Cadmiumexposition oberhalb einer Konzentration von 5 µg Cadmium/g Kreatinin im Urin eine tubuläre Nierenschädigung auftreten kann (Käfferlein 2008). Die seit 2010 publizierten Studien an Cadmium-exponierten Beschäftigten bestätigen in der Gesamtschau diesen Wert. Auch wenn die Studie von Ding et al. (2011) bei fehlender Differenzierung des Rauchverhaltens auf einen Übergang NOAEL/LOAEL bei beruflich gegen Cadmium exponierten Personen für die tubuläre Proteinurie bei ca. 3 µg Cadmium/g Kreatinin im Urin hinweist, zeigt die Studie von Chaumont et al. (2011) eine BMDL5 von 5,5 bis 6,6 µg Cadmium/g Kreatinin für die tubuläre Proteinurie für Nie-Raucher und eine niedrigere BMDL5 bei Jemals-Rauchern.

**Es wird daher ein BLW von 2 µg Cadmium/g Kreatinin festgelegt.**

Im Fließgleichgewicht besteht bezüglich des Probenahmezeitpunkts keine Beschränkung.

## Interpretation

Der international übliche Kreatininbezug ermöglicht ohne Umrechnung eine Vergleichbarkeit des BLWs mit der Studienlage. Lediglich bei alten Menschen mit reduzierter Muskelmasse und bei Kindern und Jugendlichen in der Wachstumsphase ist der Kreatininbezug gegenüber dem Volumenbezug von Nachteil; dies trifft für Beschäftigte im Erwerbsalter nicht zu.

Seit dem Jahr 2011 war der BLW für Cadmium im Urin ausgesetzt. Dies wurde mit Hinweisen auf eine Irreversibilität der Cadmium-induzierten tubulären Proteinurie, mit nephrotoxischen Effekten unterhalb des BLWs in umweltepidemiologischen Studien, und mit einer möglichen glomerulären Nierenschädigung durch Cadmium begründet. Diese Annahmen konnten wissenschaftlich nicht sicher belegt werden. Insbesondere Byber et al. (2015) konnten in ihrem systematischen Review eine Progression der cadmiumbedingten tubulären zu einer glomerulären Schädigung widerlegen. Vielmehr führt eine primär glomeruläre oder interstitielle Nierenerkrankung, z. B. eine diabetische Nephropathie oder eine Glomerulonephritis, zu einer erhöhten Ausscheidung von Mikroproteinen. Nicht nur Metallothionein, sondern auch andere Proteine binden dabei Cadmium. Umweltmedizinische, insbesondere populationsbasierte epidemiologische, Studien umfassen häufig auch ältere Menschen mit nierenbezogenen Begleiterkrankungen. Zudem liegt bei solchen Personen eine mangels Muskelmasse verminderte Kreatininausscheidung vor. Diese Phänomene verfälschen die statistische Assoziation zwischen der Cadmiumkonzentration im Urin und der Ausscheidung der Mikroproteine (sogenannte reverse causation). Zudem wird in umweltepidemiologischen Studien der Raucherstatus nur selten erfasst. Insofern sind bei fehlender Berücksichtigung solcher Confounder die umweltmedizinischen Studien für die Ableitung des BLWs ungeeignet und die mitunter berichteten Effekte bei niedrigen Cadmiumkonzentrationen nicht übertragbar. Die Studien von Roels et al. (1997), Trzcinka-Ochocka et al. (2002) und Gao et al. (2016) legen im Gegensatz zur Studie von Järup et al. (1995) eine Reversibilität der tubulären Proteinurie nach Beendigung der Cadmiumexposition nahe.

Cadmium und seine anorganischen Verbindungen wurden als gesicherte Humankanzerogene eingestuft. Hierfür ist ein lokaler Effekt cadmiumhaltiger Stäube an den Atemwegen maßgeblich. Die Einhaltung des BLWs für Cadmium im Urin bietet einen Schutz ausschließlich für Effekte des Stoffes auf die Niere. Bei einer Überschreitung des BLWs ist im Einzelfall zu prüfen, ob eine Nierenerkrankung vorliegt (z. B. bei Diabetes mellitus), die zu einer verminderten glomerulären Filtrationsrate (GFR) oder zu einer erhöhten glomerulären Proteinurie führt. In solchen Fällen sollte der Cadmiumgehalt im Blut bestimmt werden; auch der Raucherstatus ist zu beachten.
